# “I Am a Football Player and/or a Girl”: Psychosemantics of Self-Consciousness among Teenage Female Football Players

**DOI:** 10.11621/pir.2024.0303

**Published:** 2024-09-15

**Authors:** Olga G. Lopukhova

**Affiliations:** a *Kazan Federal University, Russia*; b *Kazan branch of Federal Scientific Center of Psychological and Multidisciplinary Research, Russia*

**Keywords:** self-consciousness, psychosemantic, gender self, professional self, teenage female football players, implicit conflict

## Abstract

**Background.:**

Women’s football (soccer), despite official recognition and growing popularity, is perceived as a gender-non-typical sport. This contradiction can be implicitly transmitted by society and acts as a condition of conflict in the self-consciousness of teenage female football players (TFFPs). In this study, the model of self-consciousness is defined as the semantic structures generated in the dialogic interaction “person — sociocultural environment”.

**Objective.:**

Various options, including internally conflicting ones, for the content of self-consciousness in the context of both gender (girl) and professional sports (football player) are identified in comparison with the gender personality type of TFFPs.

**Design.:**

The psychosemantic method of multiple identifications is used to assess self-consciousness in participants. Image-markers of self-identification in gender and professional sports semantics are assessed through verbal personal characteristics and non-verbal color-associative stimuli. Sample: 29 female football players aged 15 years who have been involved in pre-professional football for the last 5–7 years.

**Results.:**

In TFFPs with androgynous and masculine gender personality types, self-consciousness is not conflicted; the image of a football player has masculine semantics. In TFFPs with a feminine gender personality type, self-consciousness is not conflicted due to the transformation of the image of a football player from traditionally masculine semantics to more feminine ones. TFFPs with an undifferentiated gender personality type are characterized by contradictions at the explicit and implicit levels of self-consciousness.

**Conclusion.:**

The results confirm the concept of self-consciousness as a process of dialogical interaction between the self-image and images of the socio-cultural environment, as well as the possibility of its diagnosis as individual variants of psychosemantic structures.

## Introduction

The development of women’s football (soccer) in Russia and around the world is accelerating, and this corresponds to changing views of gender in society: the mutual penetration and mixing of activities that were traditionally considered male or female, as well as social images belonging to the categories of “masculine” and “feminine”. Plaza et al. (2017) found that sport is gendered both implicitly and explicitly, which can influence individual participation. In football, these changing views on gender and their projection in public consciousness and self-consciousness are clearly presented. Football was originally a “male” sport, and to this day, from the perspective of gender semantics, it is classified as “masculine”. Despite the long history of women’s football, researchers classify this version of professional sport as “gender-not-typical” for a woman (Artamonova & Shevchenko, 2009; Chalabaev et al., 2013; Colley et al., 2005). Moreover, according to a survey by Damadayeva (2011) on a Russian sample, football was rated as the “most unfeminine” sport. While many studies focus on the inhibiting influence of traditional gender stereotypes on the development and popularization of women’s football, and advocate for equal representation of men and women in football, the problems of developing women’s awareness of their self in professional football, in particular, self-identification as a “female footballer”, have not received due attention. Understanding the individual implicit and explicit semantics of the female football players’ self-image can be useful for developing effective methods of preventing and correcting conflictual self- consciousness in the process of their training.

### Gender Semantics of Sports

Changing concepts of gender in contemporary society are expressed in part through the fact that women are participating in activities that were previously considered the prerogative of men. Adherence to gender stereotypes means that self-realization in a gender-non-typical field of activity may result in negative consequences, including deteriorating psychological well-being and self-confidence, and irrational self-blocking of professional career opportunities (Gurieva et al., 2022). Olsen et al. (2023) found that students, in spite of their egalitarian perception of professions, show a contradictory tendency. Discrepancy in a student’s gender self-identity and their gendered perception of professional requirements can lead to decreased academic performance and increased desire to drop out. The sphere of sports and physical activity, from the perspective of gender semantic analysis, appears in the form of a complex, non-linear model, where different and sometimes opposing trends at different levels of consciousness are observed (O’Reilly et al., 2023; Plaza et al., 2017). Despite the fact that in gender semantics “sport” is considered as a masculine activity, because it represents competition, the “achievement” motivation, and intense struggle, there is a tendency towards gender division within sports. Sports that demonstrate strength and power (such as football, hockey, and basketball) are more closely associated with masculinity, compared to sports with a feminine aesthetic (such as dance, rhythmic gymnastics, and figure skating) (Chalabaev et al., 2013; Colley et al., 2005; Liu, 2023). In psychology there is a tendency to divide sports into “masculine”, “feminine”, and “gender-neutral” types (Riemer & Visio, 2003). This allows us to study the psychology of athletes in “gender-typical” and “gender-non-typical” sports. A study of gender semantics in product advertising by Nike reveals their highly masculine tone, and highlights the danger of this marginalizing influence on women participating in sports (Rasmussen et al., 2021). A study by Slater & Tiggemann (2011) found that adolescent girls involved in organized sports were more likely to experience body image ridicule from both boys and girls.

Women’s football, despite official recognition and increasing popularity, is still perceived as a gender-non-typical sport (Moreyev, 2019; Rasmussen et al., 2021), which can be implicitly and unconsciously transmitted to young football players by their environment. Research shows that in adolescence, gender differentiation of self-consciousness increases; gender stereotypes have a stronger influence on the perception of oneself and society, including sports (Boiché et al., 2014; Hill & Lynch, 1983; Meaney et al., 2002; Retelsdorf et al., 2015). At the same time, recent research has found that increased gender stereotyping in self-evaluation can be combined with more egalitarian attitudes in the evaluation of others and general beliefs (Klaczynski et al., 2020; Korlat et al., 2021). This can increase implicit conflicts in the self-consciousness of teenage female football players (TFFP). For example, it is quite typical that a TFFP, capable and promising in the opinion of her coach, leaves football without a rational understanding of her reasons for doing so. Coaches in women’s football brought up this problem and the need to prevent such unstable professional self-determination of young football players (in this case, football can be classified as an area of early sports professionalization). Theoretically, these phenomena can be explained by the specific motivation “fear of success”, conceptualized by Horner (1972). Fear of success is an unconscious conflict in self-consciousness, when a woman, at a rational level of consciousness, is focused on achieving ambitious goals and has every chance of achieving them, but unconsciously acts by blocking her opportunities (Horner, 1972). Thus, understanding the causes and content of such psychological problems requires a discussion of the explicit (rational and conscious) and implicit (unconscious and inexpressible verbally) components of self-consciousness.

### Structure and Content of Self-Consciousness

In the Russian school of psychology, Stolin (1983), while developing the activity approach of A.N. Leontyev, proposed the concept of level structures and the dialogical nature of self-consciousness. The structure of self-consciousness includes not only a cognitive, but also an emotional and value-based attitude towards oneself. Internal contradictions play a leading role in its development. Social standards are presented in consciousness, in relation to which the individual defines the self, which is manifested, in particular, in the distinction and correlation of the “Real Self” (self-understanding) and the “Ideal Self ” (generalized social images that serve as guidelines in self-development).

The level structure of self-consciousness, according to Stolin (1983), suggests that the lower level of the self-image consists of unconscious ideas, presented only in experience, and is associated with subjective, emotional attitudes about oneself. Higher-level self-consciousness is characterized as the awareness and conscious assessment of individual properties and qualities of the self. Different levels of the self-image are both functionally interconnected and relatively autonomous, which means that contradictions and conflicts can arise between them. The unit of human self-consciousness, therefore, is the “conflicting meaning of the Self,” reflecting the collision of various relationships, motives, and activities. As an individual matures, the forms and mechanisms used to identify self-consciousness are transformed and become more complex; they diverge from the factors that initially triggered them and can become conscious and controlled. In this process, complex phenomena arise that reflect such contradictions in the development of self-consciousness as “negative identification”, that is, the unconscious likening of oneself to a person towards whom the subject has a negative attitude.

The most important process underlying the development of self-consciousness is self-knowledge. This process is realized through identification and subjective differentiation, filling self-consciousness with semantic content that connects a person to other people, to culture and society. At the same time, the intra- and inter-subjectivity of a person’s self-consciousness do not oppose each other as two different realities (Znakov, 2007). In this understanding, the model of self-consciousness represents semantic structures that are generated in the dialogic interaction “person – sociocultural environment”, which correlates with the basic ideas of the ecopsychological approach to mental development (Panov, 2014).

Therefore, the measurement of self-consciousness implies a psychosemantic study of various forms of meanings (images, symbols, verbal, and non-verbal signs) in the individual consciousness, in the configuration and content of the images of the “Self-concept” (Artemyeva, 1980; Petrenko, 2013; Shmelev, 1983; Stolin, 1983). Psychosemantic methods make it possible to identify, through quantitative analysis, not only explicit, but also implicit components of self-consciousness. In addition, psychosemantic methods are focused on diagnosing dynamic, situationally determined features of self-consciousness rather than stable ones.

Contemporary ideas about the development of self-consciousness, such as the concept of dissonance between implicit and explicit beliefs (Toribio, 2021), the existence of non-conceptual and non-linguistic forms of self-apprehension (Niemeck, 2023), and the sliding scale theory of Bowins (2022), correlate with these psychosemantic methods. Thus, the methods of psychosemantics may be considered the most suitable for identifying the structure, content, and conflict manifestations of self-consciousness.

### Girls in Football: Why?

In order to investigate the causes of an athlete’s psychological problems, it is necessary to understand how the athlete identifies with the sport and how the athlete perceives the possibilities of the relationship between personal development needs and a sports career. For the purposes of this study, it is important to understand what brings a girl to football (an activity considered “doubly masculine”), how she perceives herself as a football player, and how her perception of herself as a football player is related to her perception of herself as a girl.

Various reasons have been proposed to explain the desire of girls and women to self-actualize in “masculine” activities. For example, the emergence of football as an attractive area of professionalization for girls can be explained by the general tendency of androcentrism in the public consciousness, where everything “masculine” is prioritized (Bem, 1993). It is also more common for girls who have both the ability and desire to participate in football to have the opportunity to do so without being suppressed, regardless of football’s inconsistency with the traditional female role. Kavoura et al. (2018), using a qualitative analysis of interviews with female judo athletes, described “a fighter’s identity construction” in their self-consciousness. By analogy, it can be assumed that the reason for the girl’s self-realization in football could be that she considered herself a “natural born footballer”. It is also possible that the girl’s choice of football (as a masculine sport) is facilitated by masculine tendencies of her self-consciousness. By self-actualizing in football, a masculine girl can apply and develop her masculine traits (Artamonova & Shevchenko, 2009).

Based on psychosemantic diagnostics, it was experimentally found that teenagers’ preference for gender images in television advertising is associated with their self-assessment of gender qualities, and such selectivity of perception reinforces existing tendencies in their self-consciousness (Lopukhova, 2015). In a study by Kavoura (2018), two types of identity were found among female judo athletes: “successful and feminine athlete” and “natural-born fighter”. Thus, it is possible to distinguish different options for the development of self-consciousness in athletes who participate in gender-non-typical sports: (1) self-identification through the typical characteristics of the sport, even if this contradicts gender stereotypes; (2) ignoring gender stereotypes in sports and self-perception; (3) parallel development of both gender-normative and professional sports qualities; (4) modeling and implementation of an individually adapted gender self-image in sports (for example, a new type of “female football player”), as a manifestation of the highest level of agency in self-consciousness. Clarifying these assumptions involves studying the self-consciousness of TFFPs, through both its explicit and implicit content.

The main idea of this study is that TFFPs may face the problem of combining the images of the “gender self ” and the “professional sports self ” in their self-consciousness. This assumption is outlined in the following research hypotheses:

1) the content of self-consciousness of TFFPs may be varied, including conflicting self-images in the contexts of gender (girl) and professional sports (football player);
2) conflicting and non-conflicting versions of self-consciousness may be associated with the gender personality type of these female football players.

## Methods

The diagnosis of self-consciousness is based on the psychosemantic method of multiple identifications by V.F. Petrenko (Petrenko & Mitina, 2010). This method consists of developing a structured questionnaire in which the respondent is asked to evaluate a set of images that include the image of “I am now” and other images of possible self-identification. Both verbal and non-verbal signs can serve as descriptors for evaluating images (Artemyeva, 1980; Solomin, 2001).

In accordance with the goals of this study, the following images were offered to TFFPs: “I am now”, “My idol in football”, “Ideal girl”, “Ideal guy”, and “Me in 10 years”. These serve as image-markers of self-identification in gender and professional sports semantic spaces. Two groups of descriptors were proposed for assessment: verbal personal characteristics and non-verbal color-associative stimuli. According to the concepts of level structure and dialogical structure of self-consciousness, verbal descriptors are used to diagnose conscious and socially defined self-assessment, while color association is focused on identifying emotional attitudes towards the same images (Solomin, 2001).

### Participants

The study was organized in coordination with the teachers and administration of the Miras football school in Kazan; 29 female football players aged 15 years, who had been involved in pre-professional football for the last 5–7 years, were interviewed. The age range of the sample was selected based on the premise that respondents over 15 years old can independently decide to participate in the survey, and the theory that key structures of self-consciousness (self-attitude, self-esteem, self-acceptance, self-understanding, etc.) are actively being developed as psychological neoformations at this age.

### Procedure

#### Questionnaires

The study used the questionnaire “Masculinity, femininity and the gender type of personality” (the Russian analogue of the Bem Sex Role Inventory) (Lopukhova, 2013) for two tasks. First, it was used to determine the gender personality type of the TFFPs based on their assessment of the “I am now” image. Second, when assessing images of self-identification, personal qualities from the questionnaire served as verbal descriptors. Ratings were made on a Likert scale from “1” (quality never appears) to “5” (quality always or almost always appears). The “masculinity scale”, in the Russian version of the BSRI questionnaire, includes nine qualities: courage, strong personality, assertiveness, ability to lead, willingness to take risks, dominance, masculinity, tendency to lead, strength. The “femininity scale” also includes nine qualities: compliance, shyness, a tendency to show feelings, tenderness, femininity, compassion, gentleness in statements, desire to console, friendliness. The “buffer scale” included nine gender-neutral qualities. Accordingly, 27 personal qualities were proposed as verbal descriptors for assessing self-identification images.

The nonverbal assessment of self-identification images was based on ranking the association of each image with a set of color stimuli (dark blue, blue-green, orange-red, yellow, purple, brown, black, gray) on a scale from 1 to 8, with the images rated as most to least associated with the given color.

To determine the semantic structure of self-consciousness in groups of TFFPs with different gender personality types, the acquired data was processed in accordance with the proposed procedure of the “multiple identification method” (Petrenko & Mitina, 2010). The images rated by respondents were compared using the correlation method. The resulting correlation coefficient in this method serves as an indicator of the proximity-distance of positions in the self-concept of an individual respondent or a consolidated matrix for a group of respondents. Matrices for a group of respondents were consolidated by the sum of ratings for each position. All statistical procedures were performed in MS Excel. The semantic similarity of the self-identification images proposed for evaluation is manifested in the degree of closeness of the calculated indicator to one or zero by analogy with the correlation coefficient. For example, an indicator exceeding .5 reflects the similarity (or identity) of images in self-consciousness, the proximity of the indicator to zero shows the non-identity of images, a negative value reflects the opposition of images in self-consciousness.

In this study, psychosemantic analysis of consolidated group matrices was carried out separately for TFFPs with different gender personality types (masculine, feminine, androgynous, and undifferentiated) and separately for assessment through verbal and non-verbal descriptors. Next, a comparison was made of the mutual configuration of different images of self-identification at the conscious (verbalized) and possibly unconscious (emotional-sensory) levels of consciousness. Significant differences in the mutual configuration of self-identification images at these two levels were interpreted as a probable implicit conflict in self-consciousness among TFFPs.

## Results

The results of diagnosing the gender personality type made it possible to divide the sample of 29 TFFP into four groups: 16 TFFPs with an androgynous gender personality type, five with a masculine personality type, four with a feminine personality type, and four with an undifferentiated gender personality type. Next, the configurations of self-identification images described through verbal and non-verbal descriptors were analyzed separately among TFFPs of each gender type (*see Table*). The indicators are represented as two numbers on either side of a slash (x/x). These indicators show the degree of similarity between the image of the self and semantic images at two levels of self-consciousness: assessment through verbal concepts (personal qualities), which denote generally accepted semantic categories of perception of oneself and others, and assessment through color stimuli, which are not generally accepted semantic norms, but instead reflect the individual, deep, emotional attitude about these images. Analysis of these results on generalized matrices, depending on the gender type of personality, showed similarities and differences in the configurations of self-identification images at these two levels.

**Table 1:** Indicators of Proximity in the Configuration of Self-Identification Images Assessed Through Verbal/Non-Verbal Descriptors by TFFPs with Different Gender Types of Personality

Gender types of personality	Images of self- identification	“I am now”	“My idol in football”	“Me in 10 years”	“Ideal girl”
Androgynous type (N = 16)	“My idol in football”	.39 / .53	1		
	“Me in 10 years”	.68 / .75	.55 / .64	1	
	“Ideal girl”	.43 / .56	.21 / .51	.57 / .61	1
	“Ideal guy”	.37 / .55	.62 / .51	.47 / .44	.36 / .33
Masculine type (N = 5)	“My idol in football”	.62 / .42	1		
	“Me in 10 years”	.67 / .54	.54 / .43	1	
	“Ideal girl”	.65 / .36	.51 / .34	.90 / .49	1
	“Ideal guy”	.43 / .43	.71 / .54	.51 / .49	.46 / .50
Feminine type (N = 4)	“My idol in football”	.07 / .32	1		
	“Me in 10 years”	.68 / .52	.40 / .58	1	
	“Ideal girl”	.48 / .40	.47 / .46	.68 / .11	1
	“Ideal guy”	.23 / .20	.56 / –.25	.36 / –.09	.35 / –.07
Undifferentiated type (N = 4)	“My idol in football”	.44 / .52	1		
	“Me in 10 years”	.37 / .80	.37 / .51	1	
	“Ideal girl”	.13 / .55	.13 / .03	.30 / .52	1
	“Ideal guy”	.13 / .30	.44 / .46	.32 / .18	–.09 / .14

The data presented in the Table show that for female football players with an androgynous personality type, the indicators of semantic similarity/differences of perceived images at two levels of self-consciousness do not differ greatly. In addition, female football players with an androgynous personality type do not have negative indicators that would indicate the opposition of images. The image of “I am now” is closest to the image of “Me in 10 years” (.68 / .75), as well as to the “Ideal girl” image (.43 / .56), which reflects normal gender identification. Images of gender in self-consciousness are also mostly close (.36 / .33), which corresponds to the androgynous personality type. The image of sports and professional identification (“My idol in football”) has the greatest discrepancies at the rational (verbal-evaluative) and emotional-evaluative levels of self-identification and perception of this image. In a gender context, this image, at the level of rational perception, is more masculine than feminine (closeness to “Ideal girl” .21; closeness to “Ideal guy” .62); however, at the level of emotional perception (most likely an unconscious relationship) it has no gender differentiation (closeness .51 in both cases). The perception of oneself in the future (“Me in 10 years”) is even more identified with the image of “My idol in football” (.55 / .64), which reflects an orientation towards the professional development of oneself as a football player. The image of oneself in the future is closer to the images of both male and female ideals, which also correlates with the androgynous personality type of these respondents.

In the self-consciousness of the TFFPs with a masculine type, compared to other groups, the weakest differentiation in the configuration of images proposed for evaluation is manifested. The image of “I am now” is assessed at the rational level of self-consciousness as closer to the feminine semantic (.65 with “Ideal girl”) than to the masculine semantic (.43 with “Ideal guy”), although at the level of emotional attitude, the feeling of oneself is the opposite: the self-image is closer to the masculine semantic (.43) than to the feminine (.36). This reveals to us the implicit manifestations of the masculine self-consciousness of these girls. On a rational level, masculine TFFPs also described the image of the self as quite close to the image of their ideal in football, which in their self-consciousness is presented as androgynous (close to both the male and female positions in the semantic space). At the same time, the “Ideal girl” and the “Ideal guy” in their minds are semantically similar, both at the rational and at the emotional-evaluative levels (.46 / .50), which indicates the gender undifferentiating of their self-consciousness. However, it can also be assumed that at the level of verbal assessment these girls show a tendency of “social desirability”, attributing greater compliance with normative models, while at the level of emotional attitude the picture is somewhat more complex. It seems that these “masculine” football girls really feel less like a “woman” than a “football player” does. They feel their masculinity without conflict, assessing in exactly the same way the proximity of the self-image to the masculine semantic (image of “Ideal guy”) at both levels of self-consciousness (.43 / .43).

TFFPs with a “feminine” gender type do not identify the image “I am now” with “My idol in football”, although the deep emotional relationship between these images is closer (.07 / .32). At the same time, the image of oneself in the future is much closer to the image of an idol in football (.40 / .58). It is interesting that these TFFPs also have a rather feminine image of a football player: it is close to the image of the “Ideal girl”, just like the self-image. Moreover, the image of a football player on a rational level is assessed quite close to the “Ideal guy” (.56), but is implicitly opposed (-.25). That is, the image of sports and professional self-identification is endowed with content close to their own, more feminine, self-consciousness. Gender differentiation in self-consciousness is also presented implicitly: the “Ideal girl” and the “Ideal guy” are similar in description through personality traits (.35), but in emotional and evaluative perception they are different (- .07).

TFFPs with an undifferentiated gender personality type identify themselves with the image of their “Idol in football”, both now (.44 / .52) and in the future (.37 / .51). At the same time, the image of a football player in their self-consciousness is close to masculine semantics (.44 / .46 with “Ideal guy”) and does not fit into the concept of femininity (.13 / .03 with “Ideal girl”). In a gender context, these girls do not identify their self with images of masculine or feminine semantics (.13 both with “Ideal girl” and with “Ideal guy”), which coincides with the undifferentiated gender type of their personality. However, at the emotional and evaluative level, they feel close to the image of the “Ideal girl” (.55), including in the future (.52 with “Me in 10 years”).

Thus, among TFFPs with an undifferentiated gender personality type, the greatest discrepancies between the implicit and rational levels in the perception of oneself as a girl are manifested. Self-identification with images of gender semantics is displaced by identification with the masculinized image of a professional football player, and this causes internal contradictions with the perception of oneself as a girl.

## Discussion

A comparison of the semantic context of self-identification of TFFPs of different gender types of their personality showed different tendencies in the structuring of self-consciousness at the explicit and implicit levels. The non-conflicting self-consciousness is typical for TFFPs with androgynous and masculine gender personality types. The androgynous personality type, according to the concept of Bem (1993), does not indicate internal discrepancy in the perception of “I am a football player” and “I am a girl,” since it is a manifestation of self-consciousness that is not subject to gender typing. In addition, the self-consciousness of TFFPs with a masculine personality type does not appear to be internally conflicting, since the perception of the self-image as close to the semantics of “masculine” coincides with the stereotypical perception of the “masculine” image of a football player. Girls with a masculine gender personality type apparently perceive self-realization in football, as a masculine sport, quite naturally, supporting the masculine semantics of football in self-consciousness (Artamonova & Shevchenko, 2009; Chalabaev et al., 2013; Colley et al., 2005; Damadayeva, 2011). TFFPs with a feminine gender personality type demonstrate in the content of self-consciousness a variant of transforming the image of a football player from traditionally masculine semantics to more feminine ones. TFFPs with an undifferentiated gender personality type are characterized by a discrepancy in the perception of “I am a girl” and “I am a football player,” which is especially conflicting at the implicit level of self-consciousness. Overall, these findings are consistent with the position of Plaza et al. (2017), that sport is gendered both implicitly and explicitly, which is also represented at the level of the individual consciousness of athletes, in their self-perception. This confirms the idea of a complex nonlinear understanding of consciousness, recognition of its multivariance and multi-level tendencies (O’Reilly et al., 2023).

The identified different types of relationships between the self-image and the image of a professional football player in a sample of teenage girls are similar to the results of a study by Finnish scientists (Kavoura 2018; Kavoura et al., 2018), who, using qualitative analysis methods, also revealed two different types of identity of female judokas: “successful and feminine athlete” and “natural-born fighter”.

In the present study, based on the psychosemantic method of “multiple identifications” using quantitative indicators, several variants of self-consciousness in a gender-non-typical environment of early sports professionalization (including potentially conflicting ones) were identified: (1) ignoring gender stereotypes when focusing on the image of a professional; (2) transformation of the image of a professional to suit one’s gender characteristics; (3) moving away from focusing on professional development in a gender-non-typical field of activity while maintaining a focus on gender stereotypes in self-development; (4) conflict between attitudes towards different potential images of self-identification at the explicit and implicit levels.

These data correspond to the basic concept of the model of self-consciousness, which represents semantic structures that are generated in the dialogic interaction between self-image and external images of the sociocultural environment. In the self-consciousness of the TFFP, various options for dialogical interaction “personality – socio-cultural environment” have been identified, which correlates with the positions of the ecopsychological approach to the development of the psyche (Panov, 2014), but not the influence of “marginalizing” or “masculinizing” ideas about sports and a career in football in the public consciousness (Moreev, 2019; Rasmussen et al., 2021). It should be emphasized that the data obtained in this study provide a situational picture of the dynamics of the development of self-consciousness in these girls. Moreover, the data reveal the internal mechanisms of self-consciousness structuring among female football players, but not among male football players; a difference between their semantics of sports and of gender self-identification was not discovered in the context of this study. Adolescence is a period of actualization and deepening of self-consciousness; however, it is known that girls demonstrate a higher level of both social (public) and personal self-consciousness than boys (Rankin et al., 2024). Additionally, a study by Boiché et al. (2014) found that sport–gender stereotypes are stronger in boys, whereas girls’ stereotypes increase with age. Accordingly, it can be assumed that the influence of gender ideas on self-consciousness and self-esteem changes dynamically with age.

## Conclusion

In this psychosemantic study, different conflicting and non-conflicting self-images in gender (girl) and professional sports (football player) contexts were identified in the self-consciousness of TFFPs. Female football players with an undifferentiated gender personality type perceive themselves most contradictorily as a girl and as a football player on the rational and implicit level of self-consciousness. This causes internal instability and can become a reason for leaving the sport.

In the future, research is needed to discover other possible combinations of gender and professional self-realization in the semantic structures of self-consciousness. In addition, research is required to compare the conflict in semantic structures of self-consciousness with indicators of subjective well-being and self-esteem. It is also important to explore the possibilities of psycho-corrective methods to affect the dynamics of conflicting structures and the content of self-consciousness. The present study also shows the possibilities of using the psychosemantic method of multiple identifications in the diagnostics of implicit and explicit content of self-consciousness in the work of a practical psychologist, since such a procedure for comparing images of self-identification can be carried out on a single case. Identification and awareness of the content of attitudes of self-consciousness that are hidden from understanding can provide significant assistance in counseling and psychology correction.

## Limitations

The limitations of the research results presented in this article can be divided into several aspects. Firstly, the sample is limited to late adolescence. Perhaps by studying older age groups of athletes we could obtain additional data, which could further illustrate the dynamics of self-consciousness. Secondly, this study touched only on the case of female self-realization in masculine sports; this ignores the potential differences in conflicting semantics of men’s self-consciousness when they participate in gender-non-typical sports.
